# Prediction of paclitaxel sensitivity by CDK1 and CDK2 activity in human breast cancer cells

**DOI:** 10.1186/bcr2231

**Published:** 2009-02-24

**Authors:** Satoshi Nakayama, Yasuhiro Torikoshi, Takeshi Takahashi, Tomokazu Yoshida, Tamotsu Sudo, Tomoko Matsushima, Yuko Kawasaki, Aya Katayama, Keigo Gohda, Gabriel N Hortobagyi, Shinzaburo Noguchi, Toshiyuki Sakai, Hideki Ishihara, Naoto T Ueno

**Affiliations:** 1 Central Research Laboratories, Sysmex Corporation, 4-4-4, Takatsukadai, Nishi-ku, Kobe 651-2271, Japan; 2 Breast Cancer Translational Research Laboratory, The University of Texas M. D. Anderson Cancer Center, Houston, TX 77030, USA; 3 Departments of Stem Cell Transplantation and Cellular Therapy, The University of Texas M. D. Anderson Cancer Center, Houston, TX 77030, USA; 4 Department of Breast Medical Oncology, The University of Texas M. D. Anderson Cancer Center, Houston, TX 77030, USA; 5 Department of Breast and Endocrine Surgery, Osaka University Graduate School of Medicine, 2-2 Yamada-oka, Suita-shi, Osaka 565-0871, Japan; 6 Department of Molecular-Targeting Cancer Prevention, Kyoto Prefectural University of Medicine, Kyoto 602-8566, Japan

## Abstract

**Introduction:**

Paclitaxel is used widely in the treatment of breast cancer. Not all tumors respond to this drug, however, and the characteristics that distinguish resistant tumors from sensitive tumors are not well defined. Activation of the spindle assembly checkpoint is required for paclitaxel-induced cell death. We hypothesized that cyclin-dependent kinase (CDK) 1 activity and CDK2 activity in cancer cells, which reflect the activation state of the spindle assembly checkpoint and the growth state, respectively, predict sensitivity to paclitaxel.

**Methods:**

Cell viability assays and DNA and chromatin morphology analyses were performed in human breast cancer cell lines to evaluate sensitivity to paclitaxel and the cell cycle response to paclitaxel. We then examined the specific activities of CDK1 and CDK2 in these cell lines and in xenograft models of human breast cancer before and after paclitaxel treatment. Protein expression and kinase activity of CDKs and cyclins were analyzed using a newly developed assay system.

**Results:**

In the cell lines, biological response to paclitaxel *in vitro *did not accurately predict sensitivity to paclitaxel *in vivo*. Among the breast cancer xenograft tumors, however, tumors with significantly increased CDK1 specific activity after paclitaxel treatment were sensitive to paclitaxel *in vivo*, whereas tumors without such an increase were resistant to paclitaxel *in vivo*. Baseline CDK2 specific activity was higher in tumors that were sensitive to paclitaxel than in tumors that were resistant to paclitaxel.

**Conclusions:**

The change in CDK1 specific activity of xenograft tumors after paclitaxel treatment and the CDK2 specific activity before paclitaxel treatment are both associated with the drug sensitivity *in vivo*. Analysis of cyclin-dependent kinase activity in the clinical setting could be a powerful approach for predicting paclitaxel sensitivity.

## Introduction

Paclitaxel is used widely in the treatment of breast cancer and several other solid tumors [1-6]. Paclitaxel is not effective in all tumors, however, and the characteristics that distinguish resistant tumors from sensitive tumors are not well defined. Identifying the tumor molecular characteristics associated with resistance to or sensitivity to paclitaxel would help determine which patients are most likely to benefit from paclitaxel therapy.

Paclitaxel resistance has been attributed to a variety of mechanisms, including upregulation of antiapoptotic Bcl-2 family members such as Bcl-2 and Bcl-XL [7,8]; upregulation of membrane transporters such as mdr1, which increases drug efflux [9]; point mutations in β-tubulin residues or altered expression of tubulin isotypes, which impair drug–tubulin binding [10,11]; upregulation of ErbB2 (HER-2) by inhibition of cyclin-dependent kinase (CDK) 1, which causes delayed mitosis [12]; and high expression of microtubule-associated protein tau mRNA, which decreases paclitaxel binding to microtubules and microtubule polymerization [13].

We recently reported that activation of the spindle assembly checkpoint is required for paclitaxel-induced cell death and that inactivation of this checkpoint was correlated with suppression of CDK1 activity [14]. CDK1, in combination with other mitotic cyclins, is a universal master kinase and is required for the regulation of mitosis [15]. Previous studies that used CDK inhibitors or dominant-negative CDK1 constructs showed that CDK1 plays a critical role in paclitaxel-induced cell death [16-18]. CDK1 activity may therefore be a predictor of paclitaxel sensitivity.

Several studies reported that rapidly proliferating tumors have a higher response rate to chemotherapy [19,20]. Expression levels of cyclin A and cyclin E are suggested to correlate with the proliferation state of cancer cells [21,22]. These molecules are considered to drive the cell cycle by activating CDK2 [23]. Moreover, a correlation between the effectiveness of paclitaxel and the tumor growth rate has previously been reported [24]. We therefore speculated that high CDK2 activity is required for increased paclitaxel sensitivity.

In the present study we measured both the kinase activity and expression level of CDK1 and CDK2 before and after paclitaxel treatment in human breast cancer cell lines and in xenograft models of human breast cancer. We defined the specific activity of CDK as a kinase activity (unit) of its protein expression (1 μg). We found that changes in CDK1 specific activity after paclitaxel treatment predicted the paclitaxel sensitivity of breast cancer cells and xenograft tumors. Baseline CDK2 specific activity was higher in tumors that were sensitive to paclitaxel than in tumors that were resistant to paclitaxel.

## Materials and methods

### Cell cultures

MDA-MB-231 and MDA-MB-468 human breast cancer cells were cultured in DMEM–Ham's F12. T47D and MCF-7 human breast cancer ells were cultured in RPMI 1640 medium. Both media were purchased from Sigma (St Louis, MO, USA) and were supplemented with 10% heat-inactivated FBS (Hyclone Laboratories, Logan, UT, USA) and 1% antibiotic–antimycotic solution (Gibco Invitrogen, Carlsbad, CA, USA). All cell lines were maintained at 37°C in a humidified incubator in a 5% carbon dioxide atmosphere.

### Preparation of cell lysates

Cultured cells were treated with 100 nM paclitaxel (Calbiochem, Darmstadt, Germany) for 24, 48, or 72 hours, and then these cells were frozen immediately at -80°C. At the time of CDK analysis, the frozen cells were thawed, treated with lysis buffer (0.1% NP-40, 50 mM Tris–Cl (pH 7.4), 50 mM NaF, 5 mM ethylenediamine tetraacetic acid, 1 mM NaVO_3_, and 0.2% proteinase inhibitor cocktail; Sigma), and centrifuged at 10,000 × *g *for 5 minutes at 4°C. The obtained lysates were assayed for CDK activity and expression as described below.

### Cell viability assay

For cell viability assays, cells were seeded at a concentration of 5 × 10^3 ^cells/well in 100 μl culture medium into 96-well culture plates and were incubated for 24 hours. Cells were washed, and fresh culture medium containing various concentrations of paclitaxel (0.1 pM to 100 μM) was added. After 72 hours of treatment, 10 μl WST-1/ECS solution (Chemicon International, Inc., Temecula, CA, USA) was added to each well, and the plates were incubated at 37°C for 4 hours. Absorbance was measured at 440 nm on a microplate reader (Molecular Devices, Sunnyvale, CA, USA).

### DNA analysis

For DNA analysis, cells were treated with 100 nM paclitaxel for 24, 48, or 72 hours, after which nonadherent cells were collected from the flask and then the adherent cells removed by trypsinization. The cells were stained with 50 μg/ml propidium iodide in PBS-glucose containing ribonuclease A (2 kU/ml; Sigma) for 1 hour. The DNA content of the cells was measured using a FACSCalibur flow cytometry system and was analyzed with ModFit software (BD Biosciences, San Jose, CA, USA). The DNA content was analyzed at the time corresponding to the doubling time of each cell line (that is, 24 hours after treatment for the MDA-MB-468 and MDA-MB-231 cells, and 48 hours after treatment for the T47D and MCF-7 cells).

### Chromatin morphology analysis

For chromatin morphology analysis, cells were treated with 100 nM paclitaxel for 24, 48, or 72 hours, after which nonadherent cells were collected from the flask and then the adherent cells removed by trypsinization. Both cell fractions were mixed, washed with PBS, smeared onto glass slides by cytospin centrifugation at 1,000 × *g *for 5 minutes, and then stained with aceto-orcein solution (Muto Pure Chemicals Ltd, Tokyo, Japan) to visualize the chromatin. The morphology was analyzed under a light microscope at 20× magnification. The DNA and chromatin morphology was analyzed at the time corresponding to the doubling time of each cell line after paclitaxel treatment (that is, 24 hours after treatment for MDA-MB-468 and MDA-MB-231 cells, and 48 hours after treatment for T47D and MCF-7 cells). The MDA-MB-468 and MDA-MB-231 cells could not be analyzed 48 hours after treatment because of low viability (<10%) at that time.

### Analysis of cyclin-dependent kinase and cyclin protein expression

For analysis of CDK and cyclin protein expression, the crude cell lysates containing 2.5 μg total protein were added to the wells of a newly developed dot-blot device (Sysmex Co., Kobe, Japan). The analysis procedure in detail was described in our previous report [25].

### Analysis of cyclin-dependent kinase activity

CDK molecules were selectively precipitated from 50 μg total protein with 2 μg corresponding anti-CDK1 or anti-CDK2 antibody and 20 μl protein A sepharose beads (Amersham Pharmacia, Uppsala, Sweden) for 1 hour at 4°C. Fifty microliters of the substrate mixture (containing 10 μg protein substrate (histone H1; Upstate Biotechnology, Lake Placid, NY, USA), 5 mM adenosine 5'-*O*-(3-thiotriphosphate; Sigma), 20 mM Tris–Cl (pH 7.4), and 0.1% Triton X-100) was added, and the beads were incubated under continuous shaking at 37°C for 30 minutes. The substrate mixture was then collected, and the monothiophosphates introduced as the substrate were labeled further by incubation with 10 mM iodoacetyl biotin (Pierce, Rockford, IL, USA) in coupling buffer (100 mM Tris–Cl (pH 8.5) and 1 mM ethylenediamine tetraacetic acid) for 90 minutes in the dark at room temperature. The reaction was quenched with β-mercaptoethanol, and 0.4 μg thiophosphorylated substrate was applied to the wells of the Sysmex dot-blot device. The wells were blocked with 1% bovine serum albumin for 30 minutes at room temperature, incubated with fluorescein-labeled streptavidin (Vector Laboratories, Burlingame, CA, USA) for 1 hour at 37°C, and washed with Tris-buffered saline. After that, fluorescent images of the dot-blot device membrane were evaluated with a Molecular Imager FX image analyzer (Bio-Rad, Hercules, CA, USA), and the fluorescence intensity of the dots was quantified with the Quantity One program (Bio-Rad).

### Cyclin-dependent kinase specific activity

We defined a unit as equivalent to the kinase activity of 1 μg total protein from CDK1 or CDK2.

### Xenograft models of breast cancer

MDA-MB-231 and MDA-MB-468 cells were suspended in PBS, and T47D and MCF-7 cells were suspended in a solution of Matrigel (BD, Franklin Lakes, NJ, USA) 50% v/v in PBS. The suspended cells (5 × 10^6 ^cells/mouse) were inoculated subcutaneously into the mammary fat pads of 6-week-old female BALB/c nu/nu mice (CLEA Japan Inc., Tokyo, Japan). Because T47D and MCF-7 cells require estrogen for growth, 17β-estradiol pellets (0.72 mg, 60-day release; Innovative Research of America, Sarasota, FL, USA) were implanted subcutaneously in the shoulder region of each mouse before inoculation with the cells. When the tumor masses reached 50 to 80 mm^3 ^(about 10 days after the inoculation), the mice were given daily intraperitoneal injections of paclitaxel (20 mg/kg per day, or one-half of the 50% of lethal dose 40 mg/kg,* n* = 7) or 15% ethanol solution containing 0.9% NaCl as a vehicle control (*n* = 8) for 5 days. Two-dimensional tumor measurements were made daily until 12 days after the first dose. The tumor volume was calculated according to the formula: volume = π (short diameter^2^) × (long diameter)/6.

Unpaired two-sample *t *tests were used to test differences in tumor size between the control group and the drug-treated groups. This was approved by the animal ethic committee.

The *in vivo *CDK specific activity was measured in the tumor tissues resected from the mice as follows. Tumor-bearing mice were given a single 20-mg/kg dose of paclitaxel, and 24 hours later they were killed by cervical dislocation. Tumor tissues were resected and lysed in lysis buffer with a homogenizer (HM-100; Sysmex Co.). Welch's *t *test was used for comparison.

## Results

### Sensitivity of breast cancer cell lines to paclitaxel

First, we examined the sensitivity of four human breast cancer cell lines to paclitaxel *in vitro*. The 50% inhibitory concentration (IC_50_) values determined by cell viability assay were as follows: MDA-MB-468, 1.8 nM; MDA-MB-231, 2.4 nM; T47D, 4.4 nM; and MCF-7 cells, 7.2 nM.

### Results of DNA and chromatin analysis of breast cancer cells after paclitaxel treatment *in vitro*

We next examined the cell cycle response to paclitaxel using DNA analysis and morphologic analyses of chromatin in each of the four breast cancer cell lines.

**Figure 1:**
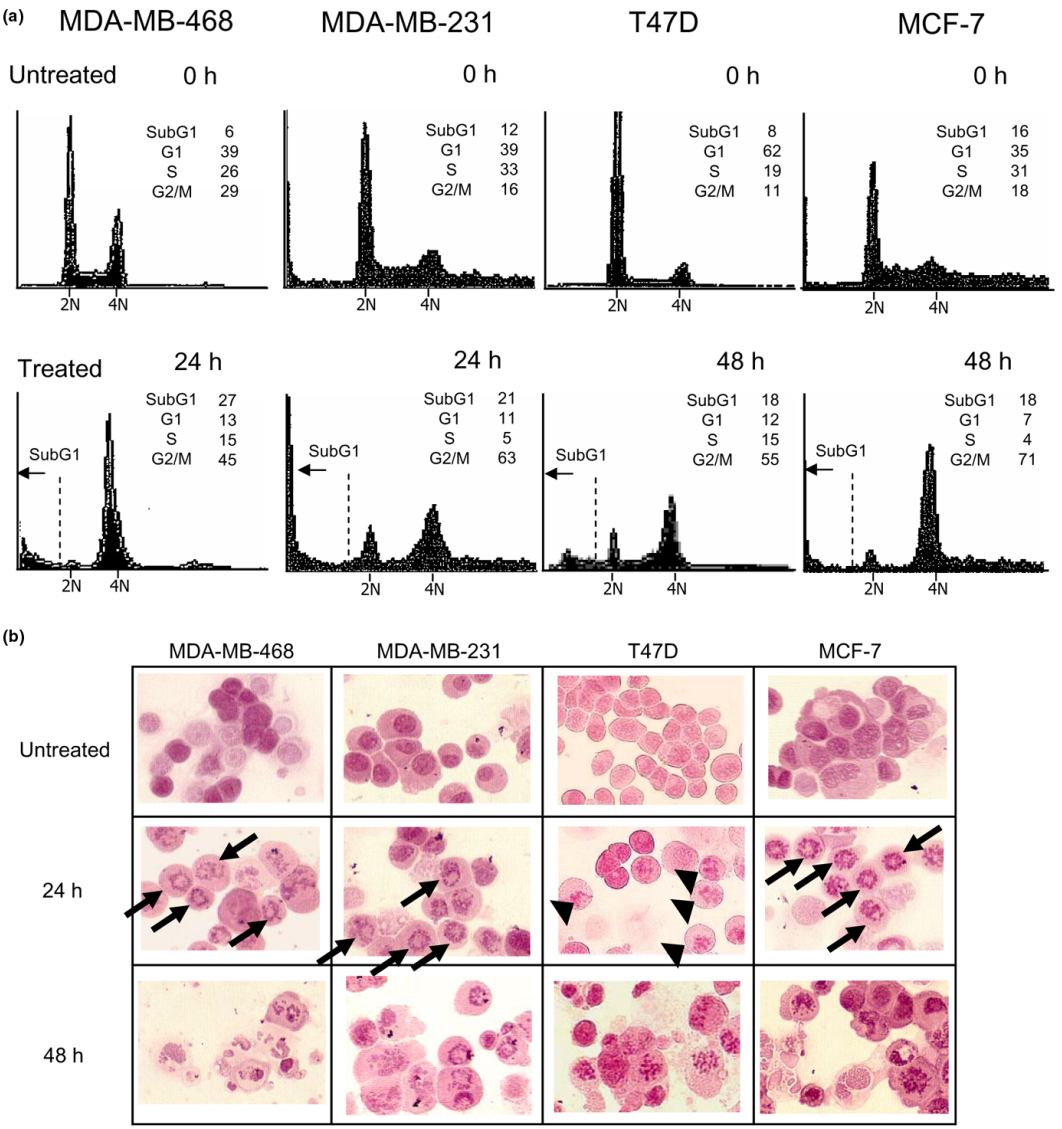
Cell cycle and morphological changes in response to paclitaxel treatment in breast cancer cell lines. Cells were treated with 100 nM paclitaxel for 0, 24, 48, or 72 hours and then stained with **(a) **propidium iodide or **(b) **aceto-orcein staining solution. Propidium iodide-stained cells were subjected to DNA analysis by flow cytometry. Morphology of orcein-stained chromatin was assessed by light microscopy. Arrowheads indicate typical chromatin condensation staining, and arrows indicate ring-like staining of mitotic cells. The flow cytometry data were analyzed at the time corresponding to the doubling time of each cell line (that is, after 24 hours of treatment for the MDA-MB-468 and MDA-MB-231 cells, and after 48 hours for the T47D and MCF-7 cells). The MDA-MB-468 and MDA-MB-231 cells could not be analyzed at 48 hours after treatment because of extremely low viability (<10%) at that time.

DNA analysis revealed that the G_2_/M (4N) fraction increased after paclitaxel treatment by a factor of 1.6 in MDA-MB-468 cells, a factor of 3.9 in MDA-MB-231 cells, a factor of 5.0 in T47D cells, and a factor of 3.9 in MCF-7 cells (Figure 1a). This result indicates that paclitaxel interfered with mitosis through spindle stabilization in each of the four cell lines. In contrast, the sub-G_1 _(apoptotic) fraction increased in three cell lines – by a factor of 4.5 in MDA-MB-468 cells, a factor of 1.8 in MDA-MB-231 cells, and a factor of 1.6 in T47D cells – but did not increase in MCF-7 cells.

In the morphologic analysis of chromatin after paclitaxel treatment, MDA-MB-468, MDA-MB-231, and MCF-7 cells showed ring-like staining (Figure 1b, arrows), indicating that paclitaxel induced perinuclear microtubule bundles [26]. T47D cells, in contrast, did not show bundles but did show chromatin condensation, which is indicative of cells arrested in mitosis (Figure 1b, arrowheads).

These results indicated that paclitaxel sensitivity *in vitro *and the cell biological response were not correlated in the four cell lines.

### Specific activities of CDK1 and CDK2 in breast cancer cells after paclitaxel treatment *in vitro*

We measured the specific activities of CDK1 and CDK2 in the four breast cancer cell lines after treatment with 100 nM paclitaxel. This concentration was chosen to ensure that cells were exposed to levels two log units higher than the IC_50 _but within the physiologically tolerable range.

**Figure 2:**
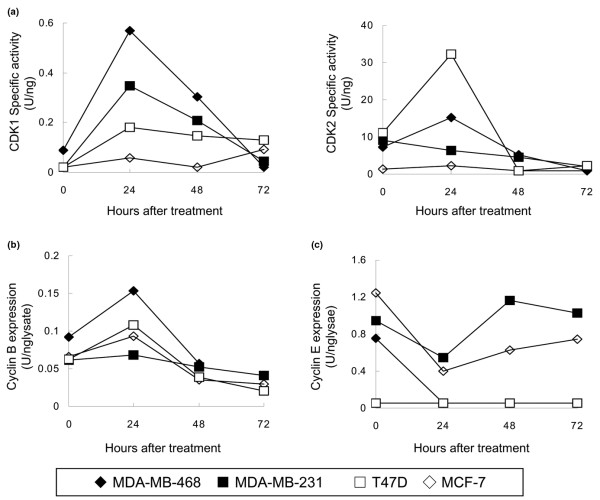
Cyclin-dependent kinase expression and specific activity levels and cyclin expression in breast cancer cell lines. Cells were treated with 100 nM paclitaxel for 0, 24, 48, or 72 hours, after which they were harvested, lysed, and assayed. **(a) **Cyclin-dependent kinase (CDK) 1 (left) and CDK2 (right) specific activity. **(b) **Cyclin B expression. **(c) **Cyclin E expression. U, units.

CDK1 specific activity was increased after paclitaxel treatment in MDA-MB-468, MDA-MB-231, and T47D cells, but not in MCF-7 cells (Figure 2a, left). The increment of CDK1 specific activity after the treatment was 6.4 times in MDA-MB-468, 8.5 times in MDA-MB-231, 4.5 times in T47D and 1.7 times in MCF-7, respectively. In contrast, CDK1 specific activity was not related to cyclin B expression after paclitaxel treatment in any of the four cell lines (Figure 2a,b). The magnitude of the increases in CDK1 specific activity after paclitaxel treatment correlated strongly with the IC_50 _values of paclitaxel in the tested cell lines (*R*^2 ^= 0.86; data not shown). These results indicated that the change in CDK1 specific activity after paclitaxel treatment reflected the sensitivity of cells to paclitaxel *in vitro*.

Similarly, CDK2 specific activity was increased after paclitaxel treatment in MDA-MB-468 and T47D cells (Figure 2a, right). The increment of CDK2 specific activity after the treatment was 2.1 times in MDA-MB-468 cells and 2.9 times in T47D cells, respectively. The magnitude of the increases in CDK2 specific activity after paclitaxel treatment, however, did not correlate significantly with the IC_50 _values of paclitaxel in the tested cell lines (*R*^2 ^= 0.004; data not shown). Cyclin E expression was not related to CDK2 specific activity after paclitaxel treatment in any of the four cell lines (Figure 2a,c).

### Sensitivity of tumor xenografts to paclitaxel

**Figure 3:**
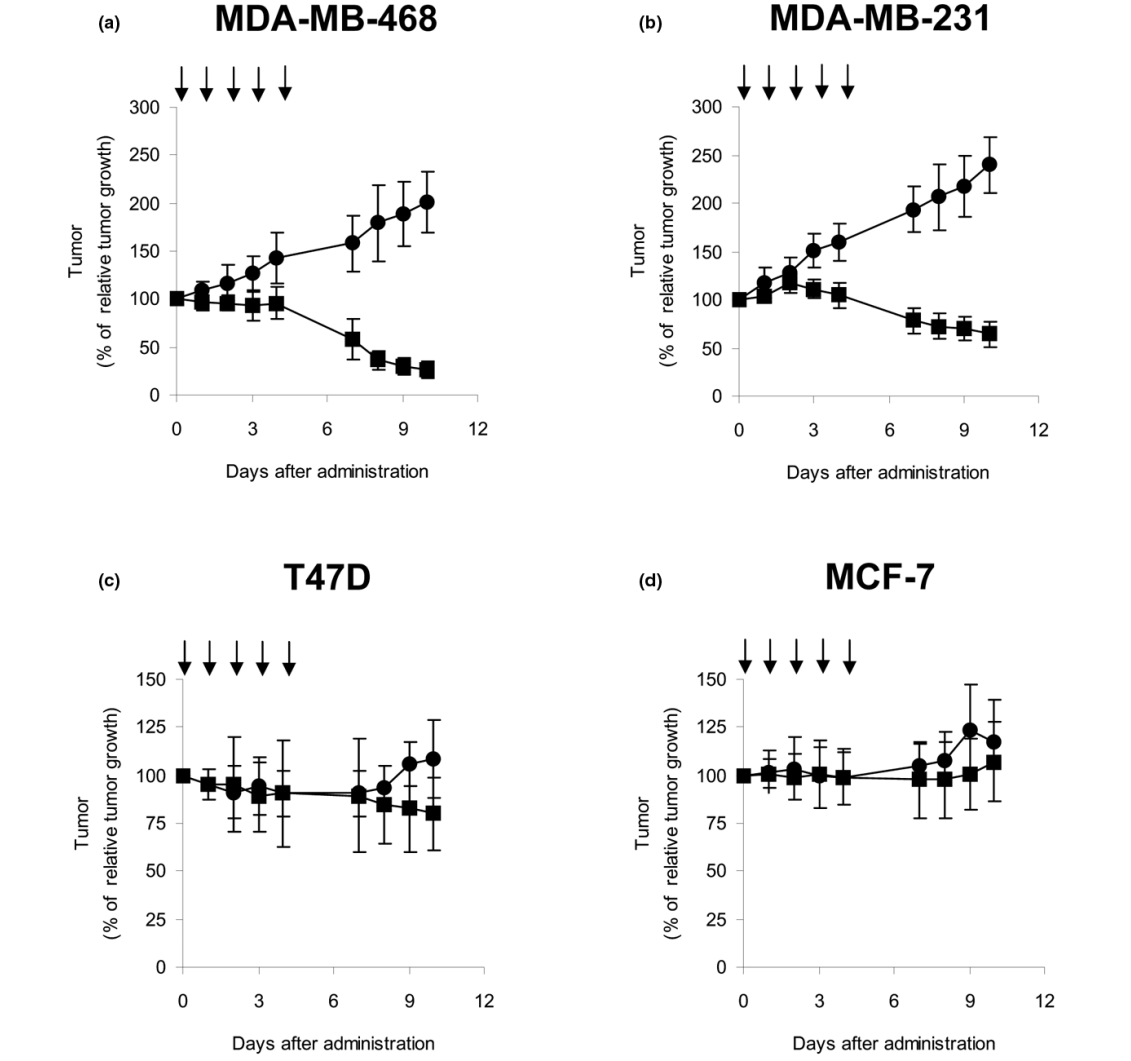
Changes in volume of human breast cancer xenograft tumors in mice after paclitaxel administration. Cells were inoculated subcutaneously into the mammary fat pads of female nude mice, and 10 days later paclitaxel (circles) or vehicle (squares) was administered daily by intraperitoneal injection for 5 days (arrows). **(a) **MDA-MB-468 cells. **(b) **MDA-MB-231 cells. **(c) **T47D cells. **(d) **MCF-7 cells. Tumor dimension was measured daily for 12 days after the first dose. Each point represents the mean (bar, standard deviation) of seven independent measurements of tumor size. Percentage of relative tumor growth calculated as the mean tumor volume on each day divided by the mean volume at the time of the first paclitaxel or vehicle administration.

We established breast cancer xenograft models by implanting each of the four breast cancer cell lines in nude mice, and then treated the mice with paclitaxel. Five daily doses of paclitaxel reduced the mean volume of the MDA-MB-468 and MDA-MB-231 tumors (Figure 3a,b) but did not affect the volume of the T47D and MCF-7 tumors (Figure 3c,d). These results indicated that, in this model, MDA-MB-468 cells and MDA-MB-231 cells were sensitive to paclitaxel and T47D cells and MCF-7 cells were resistant to paclitaxel *in vivo*.

### Specific activities of CDK1 and CDK2 in breast cancer xenografts after paclitaxel treatment *in vivo*

**Figure 4:**
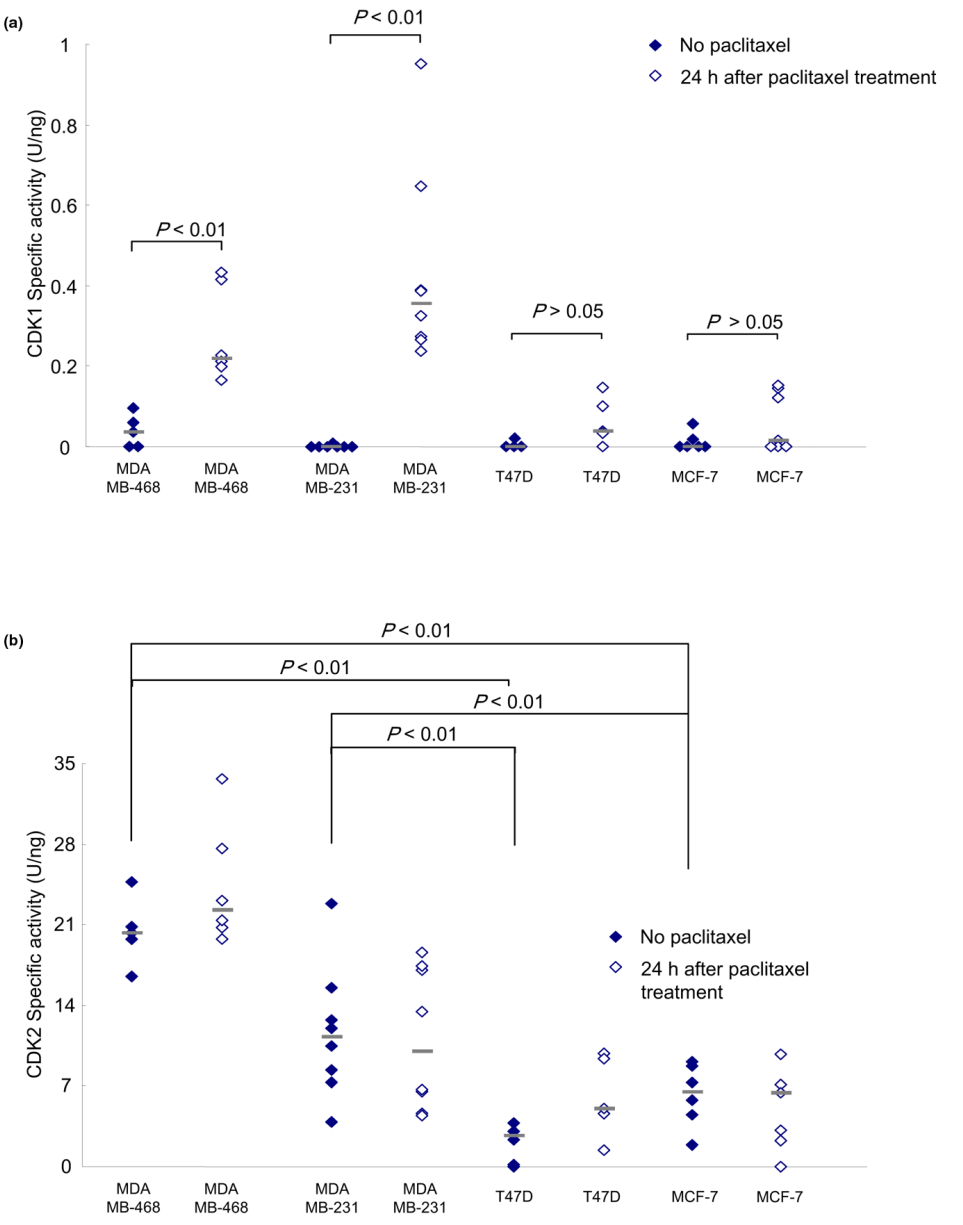
Cyclin-dependent kinase specific activity in breast cancer xenograft tissues. Previously untreated tumor-bearing mice were given a single 20-mg/kg dose of paclitaxel. Tumor tissues were resected 24 hours later, lysed, immunoprecipitated with **(a) **anti-cyclin-dependent kinase (CDK) 1 antibody or **(b) **anti-CDK2 antibody, and assayed for kinase activity. Histone H1 was used as the substrate. U, units.

We next measured the specific activities of CDK1 and CDK2 in breast tumors resected from mice 24 hours after a single dose of paclitaxel. CDK1 specific activity was significantly increased after paclitaxel treatment in the MDA-MB-468 and MDA-MB-231 tumors (Figure 4a). The mean value of CDK1 specific activity before and after the treatment was 0.036 and 0.21 units/ng in MDA-MB-468 cells, and was 0.011 and 0.35 units/ng in MDA-MB-231 cells, respectively (*P *< 0.01 for each), both of which were found to be sensitive to paclitaxel *in vivo *(Figure 3a,b); these cell lines also had the lowest IC_50 _values of paclitaxel among the four cell lines. CDK1 specific activity was not increased after paclitaxel treatment in the T47D and MCF-7 tumors (Figure 4a). The mean value of CDK1 specific activity before and after the treatment is 0.022 and 0.038 units/ng in T47D cells, and is 0.011 and 0.015 units/ng in MCF-7 cells, respectively, which were not sensitive to paclitaxel *in vivo *(Figure 3c,d).

CDK2 specific activity was increased after paclitaxel treatment only in the MDA-MB-468 tumors (Figure 4b), even though CDK2 specific activity *in vitro *was increased after paclitaxel treatment in both MDA-MB-468 and T47D cells (Figure 2a, right). Interestingly, the baseline (before paclitaxel treatment) CDK2 activity was significantly higher in the MDA-MB-468 and MDA-MB-231 tumors than in the T47D and MCF-7 tumors (Figure 4b). The mean value of CDK2 specific activity before the treatment is 20.3 units/ng in MDA-MB-468 cells, 11.2 units/ng in MDA-MB-231 cells, 2.7 in T47D cells, and 6.5 units/ng in MCF-7 cells, respectively (*P *< 0.01 for each).

## Discussion

We found that an increase in CDK1 specific activity after paclitaxel treatment correlates with sensitivity of the xenografts to paclitaxel, and a lack of change in CDK1 specific activity correlates with a lack of sensitivity of the xenografts to paclitaxel. These findings indicate that analysis of CDK1 activity could be a powerful approach for predicting paclitaxel sensitivity. In our *in vivo *experiment, the highest CDK1 specific activity value observed after paclitaxel treatment in xenografts of the paclitaxel-resistant T47D and MCF-7 cells was 0.15 units/ng. If this value was used as the cutoff value to distinguish between paclitaxel-sensitive and paclitaxel-resistant xenografts, the CDK1 specific activity would have a positive predictive value of 100% for the determination of paclitaxel sensitivity in our studies. Several reports have shown that conventional *in vitro *drug sensitivity tests such as the histoculture drug response assay are about 80% accurate in predicting sensitivity [27,28]; however, this assay is time consuming and tedious. In addition, we observed that the variation in IC_50 _values for paclitaxel determined by cell viability assay between the four cell lines was less than one order of magnitude (from 1.8 nM to 7.2 nM), which suggests that the conventional assay is difficult to apply in clinical practice. The results of our current study suggest that our newly developed system for measuring CDK activity *in vivo *would allow more accurate prediction of paclitaxel sensitivity than the conventional assay.

We reported recently that activation of the spindle assembly checkpoint is required for paclitaxel-induced cell death [14]. Assessing the function of the checkpoint in human cancer by analyzing mutation of genes or protein expression, however, would be impractical. Actually, the sensitivity of paclitaxel *in vitro *was not consistent with the cell biological response in four cell lines. MDA-MB-468 and MDA-MB-231 cells treated with paclitaxel were induced to M-phase arrest and showed ring-like staining, which was followed by apoptosis, suggesting that both cell lines possessed functional spindle assembly checkpoints. In contrast, MCF-7 cells did not show any increase in the sub-G_1 _fraction after paclitaxel treatment although they showed ring-like staining and increased in the G_2_/M fraction, indicating that the spindle assembly checkpoint – and consequent induction of apoptosis – was impaired. This response may be related to the known defect in MCF-7 cells in caspase 3 [29], which is necessary for paclitaxel-induced apoptosis [30]. This finding demonstrates that M-phase arrest in response to paclitaxel treatment does not always reflect the induction of apoptosis. In the case of T47D cells, in which no typical ring-like staining was observed, we suspect that there were point mutations in β-tubulins – including important residues for drug–tubulin binding or altered expression of tubulin isotypes. The induction of apoptosis in T47D cells upon treatment with higher concentrations of paclitaxel than the IC_50 _values may therefore depend on other checkpoints instead of, or in addition to, the spindle assembly checkpoint.

Notably, in the absence of paclitaxel treatment, the paclitaxel-sensitive MDA-MB-468 and MDA-MB-231 xenograft tumors showed much more rapid growth than the resistant T47D and MCF-7 tumors (Figure 3). Several studies reported recently that rapid proliferating tumors have a higher response rate to chemotherapy [19,20]. Actually, we found that the rapidly growing tumors showed significantly higher CDK2 specific activity without paclitaxel treatment than did the slowly growing tumors (Figure 4b). Moreover, a correlation between the effectiveness of paclitaxel and the tumor growth rate has previously been reported [24]. CDK2 specific activity in breast tumors would therefore be another indicator of paclitaxel sensitivity.

Consequently, accurate prediction could be expected from the combination assay of CDK1 and CDK2 activities. A clinical study is needed to validate our concept of predicting sensitivity to paclitaxel by analyzing the CDK activity in tumor tissues from patients.

## Conclusions

The increase in CDK1 specific activity after paclitaxel treatment indicates that a tumor is sensitive to paclitaxel, and a lack of change in CDK1 indicates that a tumor is resistant to paclitaxel. The level of CDK2 specific activity before paclitaxel treatment was shown to correlate with paclitaxel sensitivity *in vivo*. Consequently, accurate prediction of paclitaxel sensitivity could be realized by a combination assay of CDK1 and CDK2 activities. Validation of our concept with a clinical sample will be needed in future studies.

## Abbreviations

CDK: cyclin-dependent kinase; DMEM: Dulbecco's modified Eagle's medium; FBS: fetal bovine serum; IC_50_: 50% inhibitory concentration; PBS: phosphate-buffered saline.

## Competing interests

SNa, YT, TY, TM, YK, AK, KG, and HI are employed by Sysmex Corporation. Sysmex Corporation supports the projects of SNo, TSa and NTU. The other authors declare that they have no competing interests.

## Authors' contributions

SNa and YT participated in the design of the study, carried out many of the experiments, and drafted the manuscript. TT and TSu participated in the design of the study and data interpretation. KG, TY and TM participated in the design of the study and helped draft the manuscript. YK and AK carried out the protein expression and kinase analyses and performed the statistical analyses. GNH, SNo, and TSa participated in data interpretation and gave critical suggestions. HI and NTU led the conception and design of the study and the revisions to the manuscript and supervised this research project. All authors read and approved the final manuscript.
